# Characterization of *Pseudomonas capsici* strains from pepper and tomato

**DOI:** 10.3389/fmicb.2023.1267395

**Published:** 2023-10-11

**Authors:** Mei Zhao, Ron Gitaitis, Bhabesh Dutta

**Affiliations:** ^1^Department of Plant Pathology, College of Plant Protection, China Agricultural University, Beijing, China; ^2^Department of Plant Pathology, University of Georgia, Tifton, GA, United States

**Keywords:** *Pseudomonas capsici*, ANI, dDDH, copper, host range, effector

## Abstract

Disease outbreaks of bacterial leaf spot and blight of pepper and tomato often occur in both transplant- and field-production systems worldwide. In some cases, the outbreaks are caused by novel bacterial species. Characterization of these novel bacterial species are critical in developing diagnostic assays and identifying management options for pathogen monitoring and sustainable production, respectively. We characterized strains belonging to novel *Pseudomonas* species that are responsible for outbreaks in pepper and tomato both in transplant-houses and in production fields in Georgia, USA. Phylogenomic analyses and whole genome sequence indices demonstrated that the pepper and tomato strains belonged to *P. capsici*. The whole-genome comparison revealed that 13 *Pseudomonas* strains from diverse isolation sources that were curated in NCBI were indeed *P. capsici* indicating a potential wide-host range for this bacterial species. Our greenhouse-based host-range assay also indicated that *P. capsici* strains were pathogenic on pepper, tomato, eggplant, cabbage, lettuce, and watermelon corroborating a wide-host-range. A phylogenetic tree inferred from the whole genome sequence data showed that the *P. capsici* strains from Georgia (pepper and tomato) were genetically diverse, and were closely related to tomato *P. capsici* strains from Florida. Genomic presence of traditional bacterial virulence factors in *P. capsici* strains was also determined. *Pseudomonascapsici* strains encode one set of type I secretion system, two sets of type II secretion systems, one set of type III secretion system, two sets of type V secretion systems, three sets of type VI secretion systems, and various secondary metabolite gene clusters including lipopeptides. In *in-vitro* assays, it was demonstrated that six out of seven *P. capsici* strains (pepper and tomato strains from Georgia) were not sensitive to 0.8 mM CuSO_4_. When the genomes of copper-tolerant strains were compared with the copper-sensitive strains, it was observed that the former strains encode a cluster of genes related to copper tolerance, which were absent in the genomes of copper-sensitive strains. Considering the ability of *P. capsici* strains to infect a range of vegetable hosts and possession of a wide range of bacterial virulence factors, secondary metabolites, and copper-tolerance genes, we envision that the management of this pathogen might potentially be a challenge.

## 1. Introduction

Tomato (*Solanum lycopersicum*) and pepper (*Capsicum annuum*) are important vegetable crops in the United States. However, they are vulnerable to several *Pseudomonas* species, including *P. corrugata*, *P. syringae* pv. *tomato,* and *P. syringae* pv*. syringae*, *P. viridiflava*, *P. mediterranea,* which have been known to cause diseases in these crops in the United States particularly in Georgia and Florida (Jones et al., 1981; McCarter et al., 1983; Bonn et al., 1985; Jones et al., 1986; Gitaitis et al., 1987; Voloudakis et al., 1991; Searcy et al., 2015). In fact, *P. syringae* pv. *syringae* and *P. syringae* pv. *tomato* strains were routinely recovered from field-grown tomato transplants in Georgia (Gitaitis et al., 1985). Later, the number of outbreaks reduced considerably when the transplant production was shifted completely inside temperature-controlled greenhouses. Nevertheless, sporadic outbreaks on a limited scale still occur in the greenhouse production systems.

In August 2020, two separate bacterial disease outbreaks in tomato were reported in Georgia, USA, one in the greenhouse and another in the commercial production field. In both outbreaks, the affected foliage displayed irregular lesions with distinct yellow margins. Upon bacterial isolation from symptomatic foliage and 16S rRNA sequence analysis, the strains were found to be closely related to *P. cichorii*. The strains were positive for oxidase activity, were able to rot potato, and were also able to induce a hypersensitive reaction on tobacco leaves. However, the strains were negative for levan production and arginine dihydrolase utilization. It is worth noting that these tomato strains were able to rot potatoes, which was different from the typical *P. cichorii* LOPAT profile (− + −−+) (Lelliott et al., 1966).

We previously reported a new *Pseudomonas* species, *P. capsici,* that caused typical bacterial symptoms on pepper foliage (leaf spots and blights) under greenhouse conditions (Zhao et al., 2021). The pathogen was also able to cause lesions on fruit that turned necrotic and eventually resulted in fruit rot in pepper. *Pseudomonas capsici* is closely related to *P. cichorii*, and strains from pepper could also rot potatoes (Zhao et al., 2021). In this manuscript, we determined that tomato strains isolated from two separate outbreaks were *P. capsici,* not *P. cichorii*. Furthermore, we also provided a detailed characterization of these tomato and pepper strains from recent outbreaks in Georgia, United States. The characterization included genome analysis for relatedness and the presence of traditional virulence factors, evaluation of host-range under greenhouse conditions, and copper tolerance (phenotypic and genotypic evidence). These results will provide insights into the molecular mechanisms underlying *P. capsici* virulence, and may prove useful in developing new strategies for managing this destructive pathogen.

## 2. Materials and methods

### 2.1. Bacterial isolation and inoculum preparation

In 2020, tomato foliage samples exhibiting symptoms were collected from Cook County and Grady County in Georgia, United States. For bacterial isolation, the margins of healthy and symptomatic tomato leaf tissues were excised using a sterile scalpel and then macerated in sterile distilled water (sdH_2_O). The resulting tissue macerates were then streaked onto the nutrient agar (NA) medium. The NA plates were incubated at 28°C for 2 days. The dominant colonies on the isolation plates were selected and streaked onto new NA plates to obtain pure cultures. In addition, three *P. capsici* strains (Pc19-1^T^, Pc19-2, and Pc19-3) isolated from pepper in Georgia (Zhao et al., 2021), two *Pseudomonas* strains (GEV417 and GEV1127) isolated from tomato in Florida (Timilsina et al., 2017), and three *Pseudomonas* strains (NCPPB1511, NCPPB2479, and NCPPB3928) obtained from the National Collection of Plant Pathogenic Bacteria (NCPPB) were also included and characterized in this study. The location, host, and year of isolations for these strains are shown in Table 1.

**Table 1:** List of *Pseudomonas capsici* strains characterized in this study, isolation location, host, and year.

Strain	Isolation location	Isolation host	Isolation year
Pc19-1^T^	Colquitt County, Georgia	Pepper	2019
Pc19-2	Colquitt County, Georgia	Pepper	2019
Pc19-3	Colquitt County, Georgia	Pepper	2019
Pc20-2	Cook County, Georgia	Tomato	2020
Pc20-3	Grady County, Georgia	Tomato	2020
Pc20-4	Grady County, Georgia	Tomato	2020
Pc20-5	Grady County, Georgia	Tomato	2020
GEV417	Florida	Tomato	2011
GEV1127	Florida	Tomato	2012
NCPPB1511	USA	Cabbage	1963
NCPPB2479	Barbados	Lettuce	1972
NCPPB3928	Brazil	Chinese cabbage	1995

The *Pseudomonas* strains were routinely cultured on NA at 28°C for 2 days. To prepare the inoculum, the strains were grown in nutrient broth in a shaking incubator (MaxQ 4,450, Thermo Scientific, Waltham, MA) at 28°C and 200 rpm for around 16 h. After centrifugation at 16,100 x g for 1 min, the supernatants were then removed, and the resulting pellets were resuspended in sdH_2_O. The bacterial concentrations were then adjusted to an optical density of 0.3 at 600 nm, which corresponds to approximately 10^8^ colony-forming units (CFU/mL), using a Biophotometer (Eppendorf, Hamburg, Germany).

### 2.2. Pathogen identification

#### 2.2.1. Genome sequencing and assembly

In order to identify the species of bacterial isolates, we utilized whole genome sequencing on the twelve *Pseudomonas* strains. First, single colonies from each strain were transferred from NA plates to 4 mL nutrient broth and cultured overnight in a shaking incubator (200 rpm, 28°C). Then, a Monarch Genomic DNA Purification Kit (New England Biolabs, Ipswich, MA) was used to extract genomic DNA from 1 mL of overnight culture. A NEBNext Ultra II DNA Library Prep Kit for Illumina was used to prepare genomic libraries and the Illumina Novaseq 6,000 platform was used to sequence the libraries by Novogene Co., Ltd. (Beijing, China). Raw sequences were filtered using fastp v 0.20.0 (Chen et al., 2018), and quality checks were conducted using fastqc v 0.11.9^1^. The processed reads were assembled using SPAdes v 3.14 (−-isolate --cov-cutoff auto mode) (Bankevich et al., 2012) and filtered for a minimum contig size of 500 bp. The final assemblies were deposited in the NCBI database under the BioProject PRJNA890938, and uploaded to the Life Identification Number (LIN) platform developed by Tian et al. (2020).

#### 2.2.2. Digital DNA–DNA hybridization (dDDH) and average nucleotide identity (ANI)

To determine the taxonomic classification of the twelve *Pseudomonas* strains at the species level, we conducted a comparative analysis of their dDDH and ANI values with the *P. capsici* type strain Pc19-1^T^. For dDDH values, the recommended settings [formula 2; i.e. GBDP formula d4; sum of all identities found in HSPs (high-scoring segment pairs) divided by overall HSP length] of the genome-to-genome distance calculator 2.1 (Meier-Kolthoff et al., 2013) were utilized for calculation using the Type Strain Genomic Server (TYGS) (Meier-Kolthoff and Göker, 2019). Additionally, the ANI values based on the BLAST algorithm (ANIb) were calculated using jSpeciesWS v1.2.1 (Richter et al., 2016).

#### 2.2.3. Phylogenomic analysis

The species identity of all 49 *P. cichorii* and 12 *P. capsici* strains with genome assemblies that were available on NCBI (accessed on Dec 07 2022) were screened using the TYGS. Based on the results, strains that were confirmed as *P. capsici,* along with *P. cichorii* ATCC 10857^T^ (RefSeq assembly accession: GCF_900104015.1) as an outgroup, were chosen for phylogenetic analysis based on their core genomes using the M1CR0B1AL1Z3R web server^2^ (Avram et al., 2019). The default settings (maximal e-value cutoff: 0.01, identity minimal percent cutoff: 80.0%, minimal percentage for core: 100.0%) were employed. Bootstrap analyses were enabled to increase the statistical robustness of the results. Similarly, phylogenomic analysis of the 12 *P. capsici* strains in Table 1 was also conducted using the M1CR0B1AL1Z3R web server with the default settings and bootstrap analyses enabled. *Pseudomonas cichorii* ATCC 10857^T^ was included as an outgroup.

#### 2.2.4. Phylogenetic analysis of housekeeping genes *gyrB* and *rpoD*

The sequences of housekeeping genes *gyrB* and *rpoD* were extracted from the genomes of 12 *P. capsici* strains and selected strains using BLASTN and analyzed in Geneious Prime (v2019.2.3). The individual gene sequences were aligned using MAFFT (v7.294b) (Katoh and Standley, 2013) and trimmed. The concatenated alignments of *gyrB* (487 bp) and *rpoD* (474 bp) were used to construct a neighbor-joining phylogenetic tree. The robustness of the tree topology was estimated using 1,000 bootstrap replicates. The tree was visualized using the tvBOT (Xie et al., 2023).

### 2.3. Host range assays

The host range assays were performed on pepper cv. Aristotle, tomato cv. Glacier, Chinese cabbage (*Brassica rapa* ssp*. chinensis*) cv. Rubicon, eggplant (*Solanum melongena*) cv. Nadia, lettuce (*Lactuca sativa*) cv. Dragoon, broccoli (*Brassica oleracea* var. *italica*) cv. Arcadia F1, and endive (*Cichorium endivia*) cv. Curlesi. Seedlings (*n* = 10 seedlings per host per experiment) were grown in plastic pots filled with commercial potting mix and maintained at a temperature of 28°C in a greenhouse. Two independent experiments were conducted. To inoculate the seedlings, leaves of four to six-week-old seedlings of each host were infiltrated with bacterial suspensions of *Pseudomonas* strains in Table 1 at a concentration of 10^6^ CFU/mL using a syringe. Seedlings inoculated with sdH_2_O were used as negative controls. The symptom was evaluated qualitatively (presence/absence) in the inoculated seedlings at 7 days post-inoculation (dpi). The bacteria were re-isolated from the symptomatic tissues and the identities of the isolated bacteria were confirmed by BOX-PCR (Brusetti et al., 2008). Briefly, isolated bacterial strains were inoculated into 3 mL of nutrient broth and incubated on a rotary shaker (Innova; New Brunswick Scientific Co., Edison, NJ) at 250 rpm for 18 h. After incubation, cells were harvested by centrifugation at 6,000 × g (Allegra 25R, Beckman Coulter, Fullerton, CA) for 5 min and DNA was extracted using the UltraClean Microbial DNA Kit (MO BIO, Carlsbad, CA) according to the manufacturer’s instructions. For BOX-PCR, 2 μL of bacterial DNA were amplified using 10 μM of BOXA1R primer (5’-CTA CGG CAA GGC GAC GCT GAC G-3′) according to PCR conditions as described previously (Brusetti et al., 2008). PCR products (10 μL) were separated by electrophoresis at 125 V for 4 h on a 1.5% agarose gel in 1X Trisborate ethylenediaminetetraacetic acid (EDTA) buffer.

### 2.4. Copper tolerance tests

Nutrient agar supplemented with 0.8 mM copper sulphate (CuSO_4_· 5H_2_O) was prepared following published protocols (Walcott et al., 2004; Stice et al., 2018). Ten microliters of each 1 × 10^8^ CFU/mL bacterial suspension in sdH_2_O were spotted onto the plates, and growth was visually evaluated and recorded as positive or negative after two days of incubation at 28°C. *Acidovorax citrulli* group I strain M6, and group II strain AAC00-1 were used as copper-tolerant and sensitive controls, respectively. Each strain was tested in triplicate, and the experiment was conducted twice.

### 2.5. Computational identification of unique copper tolerance genes, protein secretion systems, and secondary metabolite gene clusters

Genome annotation was performed using Rapid Annotation using Subsystem Technology (RAST v2.0) server (Overbeek et al., 2014). The subsystems of the annotated genomes related to copper were further manually reviewed on the RAST server. Copper tolerance gene clusters unique to the copper-tolerant strains were identified and their nucleotide sequences were extracted and aligned using MAFFT in Geneious. Protein secretion systems were predicted using TXSScan (Abby et al., 2014, 2016). Operon maps of the identified copper tolerance clusters and protein secretion systems were generated using Gene Graphics (Harrison et al., 2018) and annotated in PowerPoint. The assembly files were used as input data for *in silico* secondary metabolite gene cluster analysis using antiSMASH v 6 with default parameters and the ‘knownclusterblast’ flag (Blin et al., 2021).

## 3. Results

### 3.1. General features of the genome sequences

The general features of the 12 *Pseudomonas* sequenced genomes are summarized in Table 2. The genome lengths of the assemblies ranged from 5.82 Mbp (NCPPB2479) to 6.05 Mbp (NCPPB1511) with an average length of 5.91 Mb. The number of contigs in each genome assembly ranged from 40 to 59. The N50 of the assemblies ranged from 201,021 bp (GEV417) to 362,351 bp (NCPPB3928). The total number of genes in each genome ranges from 5,104 (Pc19-2) to 5,367 (NCPPB1511). The predicted total number of protein-coding genes varies from 4,991 (Pc19-2) to 5,248 (NCPPB1511). The GC content of the twelve genomes ranges from 58.37 to 58.58%, with an average of 58.43% (Table 2). The LINs for the twelve *Pseudomonas* genomes are shown in Table 3.

**Table 2:** Genomic characteristics of *Pseudomonas capsici* strains.

Strain	Total length	Contig numbers	N50^a^	Total gene	Total protein-coding gene	GC content	Assembly Accession	BioProject Accession	BioSample Accession
Pc19-1^T^	5,843,696	59	332,309	5,105	4,992	58.43	GCA_017165765.1	PRJNA700700	SAMN17837728
Pc19-2	5,846,317	55	241,172	5,104	4,991	58.43	GCA_017165785.1	PRJNA700775	SAMN17838719
Pc19-3	5,845,043	59	205,290	5,105	4,996	58.43	GCA_017165745.1	PRJNA700776	SAMN17838721
Pc20-2	5,909,053	53	338,792	5,138	5,018	58.50	GCA_025791895.1	PRJNA890938	SAMN31305587
Pc20-3	5,909,484	58	274,281	5,182	5,062	58.37	GCA_025792395.1	PRJNA890938	SAMN31305588
Pc20-4	5,908,954	53	300,467	5,180	5,060	58.37	GCA_025791915.1	PRJNA890938	SAMN31305589
Pc20-5	5,909,119	59	231,715	5,179	5,063	58.37	GCA_025792375.1	PRJNA890938	SAMN31305590
GEV417	5,954,550	52	201,021	5,198	5,080	58.44	GCA_025791925.1	PRJNA890938	SAMN31305585
GEV1127	5,909,743	57	298,636	5,173	5,056	58.37	GCA_025791905.1	PRJNA890938	SAMN31305586
NCPPB1511	6,053,098	44	273,088	5,367	5,248	58.41	GCA_025792135.1	PRJNA890938	SAMN31305582
NCPPB2479	5,822,782	45	218,322	5,128	5,031	58.50	GCA_025792415.1	PRJNA890938	SAMN31305583
NCPPB3928	5,953,896	40	362,351	5,251	5,126	58.58	GCA_025791975.1	PRJNA890938	SAMN31305584

a N50 is the contig length such that using longer or equal length contigs produces half of the bases of the assembly.

**Table 3:** Life Identification Number (LIN)^a^ of *Pseudomonas capsici* strains.

	70%	75%	80%	85%	90%	95%	96%	97%	98%	98.5%	99%	99.25%	99.5%	99.75%	99.9%	99.925%	99.95%	99.975%	99.99%	99.999%
Strain	A	B	C	D	E	F	G	H	I	J	K	L	M	N	O	P	Q	R	S	T
Pc19-1^T^	50	1	0	2	0	0	0	0	0	0	0	0	0	0	0	0	0	0	0	0
Pc19-2	50	1	0	2	0	0	0	0	0	0	0	0	0	0	0	0	0	0	0	1
Pc19-3	50	1	0	2	0	0	0	0	0	0	0	0	0	0	0	0	0	0	0	2
Pc20-2	50	1	0	2	0	0	0	0	0	0	4	0	0	0	0	0	0	0	0	0
Pc20-3	50	1	0	2	0	0	0	0	0	0	0	0	0	1	0	0	0	0	0	1
Pc20-4	50	1	0	2	0	0	0	0	0	0	0	0	0	1	0	0	0	0	0	2
Pc20-5	50	1	0	2	0	0	0	0	0	0	0	0	0	1	0	0	0	0	0	3
GEV1127	50	1	0	2	0	0	0	0	0	0	0	0	0	1	0	0	0	0	0	0
GEV417	50	1	0	2	0	0	0	0	0	0	2	0	0	0	0	0	0	0	0	0
NCPPB1511	50	1	0	2	0	0	0	0	0	0	3	0	0	0	0	0	0	0	0	0
NCPPB2479	50	1	0	2	0	0	0	0	1	0	0	0	0	0	0	0	0	0	0	0
NCPPB3928	50	1	0	2	0	0	0	0	2	0	0	0	0	0	0	0	0	0	0	0

a LIN is assigned to each genome based on its similarity with the closest genome in the database measured as percentage of average nucleotide identity (ANI). The number at each LIN position (A–T) are used as symbols for different ANI thresholds.

### 3.2. Pathogen identification

To determine the species identity of the 12 *Pseudomonas* genomes, their dDDH and ANIb values were calculated by comparing them with the *P. capsici* type strain Pc19-1^T^. All strains displayed dDDH values ranging from 77.0 to 100.0% when compared with *P. capsici* Pc19-1^T^, exceeding the cut-off value of 70% for species delineation based on dDDH (Table 4). Similarly, all strains exhibited ANIb values ranging from 97.1 to 100.0% when compared with *P. capsici* Pc19-1^T^, surpassing the commonly accepted threshold of 95–96% for species delineation based on ANIb (Table 4). These observations indicate that all twelve strains belonged to the same species. Furthermore, 13 genomes listed under the *P. cichorii* genome list in the NCBI database had dDDH values exceeding 70%, indicating that they are in fact *P. capsici.* The list of strains that were wrongly speciated as *P. cichorri* in the NCBI database but were indeed *P. capsici* based on the above genome-based assays include; 473, MAFF 302698, Ku1409-10-1, NB15027, LCDW06, LCFQ22, 481, 482, 136, 474, WSXC14, s-2-2-1, and ICMP 1649.

**Table 4:** Genomic relationship between *Pseudomonas capsici* strains and the type strain of *Pseudomonas capsici* Pc19-1^T^.

Query strain (*P. capsici*)	Pc19-1^T^ dDDH (%)	Pc19-1^T^ ANIb (%)
Pc19-2	100.0	100.0
Pc19-3	100.0	100.0
Pc20-2	87.4	98.4
Pc20-3	96.6	99.5
Pc20-4	96.6	99.5
Pc20-5	96.6	99.5
GEV417	88.0	98.4
GEV1127	96.6	99.5
NCPPB1511	87.9	98.4
NCPPB2479	76.5	97.0
NCPPB3928	77.0	97.1

Digital DNA–DNA hybridization (dDDH) values and average nucleotide identity (ANI) were calculated using Genome-to-Genome Distance Calculator 2.1 (formula 2), and JSpeciesWS v1.2.1, respectively.

**Figure 1:**
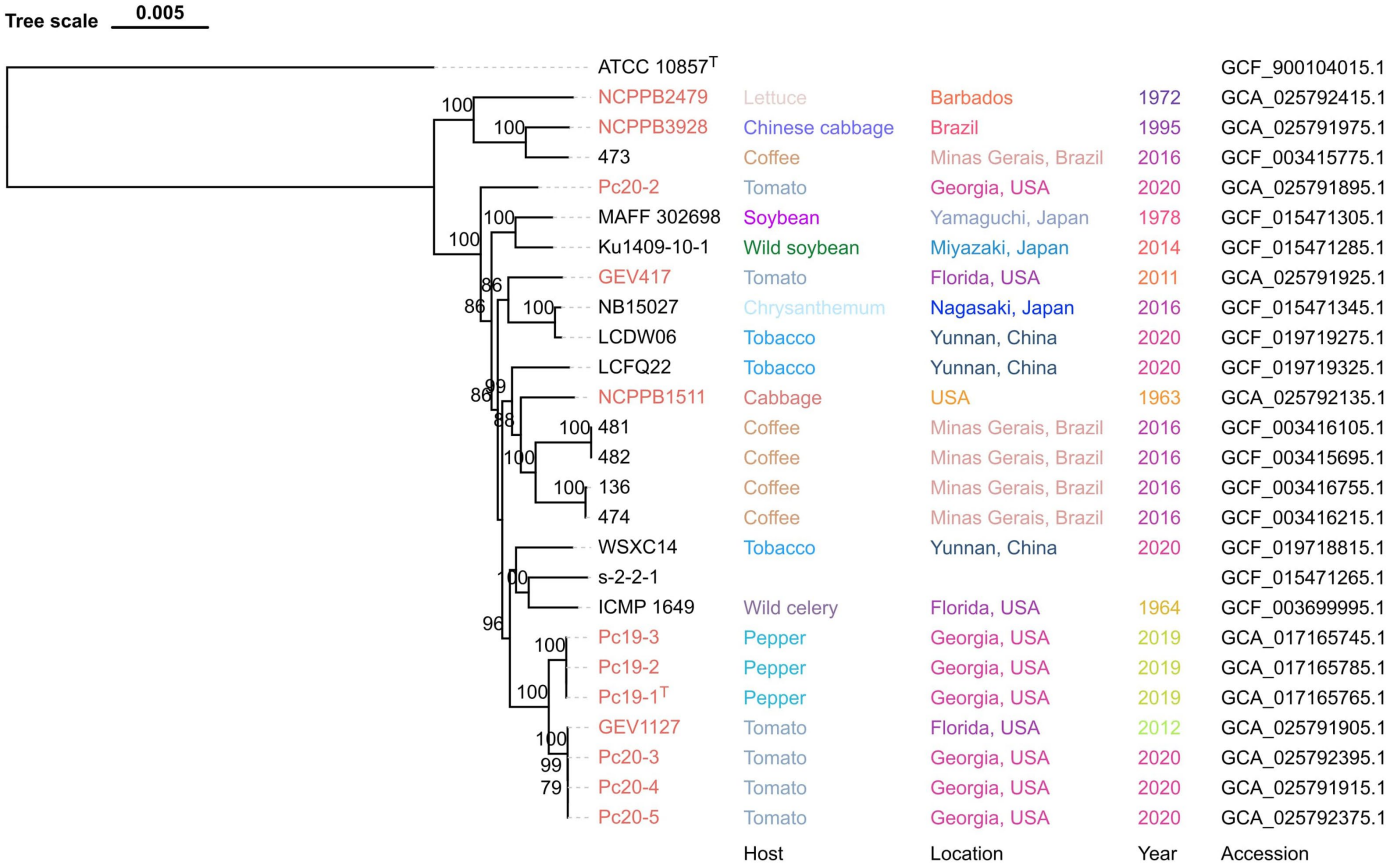
Maximum-likelihood phylogeny of *Pseudomonas capsici* strains publicly available based on whole genome sequences. *Pseudomonas cichorii* ATCC 10857^T^ was used as an outgroup. *Pseudomonas capsici* strains characterized in this study are labeled in red. Bootstrap values >70% are shown as percentages of 1,000 replicates. The scale bar represents nucleotide substitutions per site. Isolation host, location, year, and assembly accession are shown.

The phylogenomic tree, which was generated using the M1CR0B1AL1Z3R web server, is based on the alignment of the core-proteome (4,216 genes) of all 25 *P. capsici* strains, with *P. cichorii* ATCC 10857^T^ as an outgroup. This core-proteome tree showed that the Georgia pepper strains Pc19-1^T^, Pc19-2, and Pc19-3 formed a cluster that was closely related to another cluster consisting of Georgia tomato strains Pc20-3, Pc20-4, Pc20-5, and a tomato strain from Florida GEV1127, with 100% support values (Figure 1). These observations suggest that these strains have a close evolutionary relationship with each other. However, strain Pc20-2, which was isolated from a separate tomato outbreak in Cook County, Georgia, was found to be distinct from other strains, indicating a unique source of introduction and outbreak (Figure 1).

### 3.3. Phylogenetic analysis based on *gyrB* and *rpoD* genes

**Figure 2:**
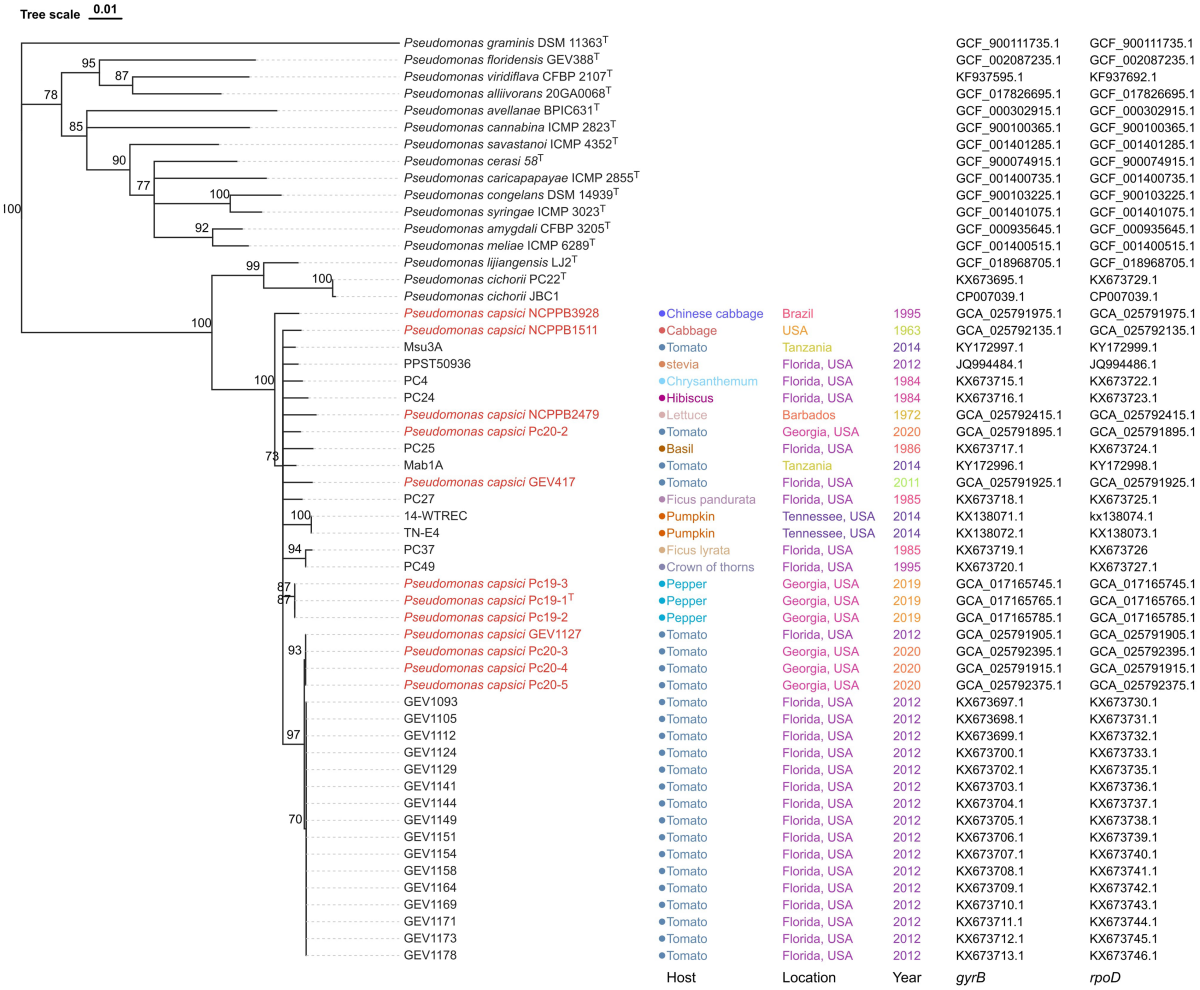
Neighbor-joining phylogeny of *Pseudomonas* strains using concatenated sequences of *gyrB* and *rpoD* genes. *Pseudomonas graminis* DSM 11363^T^ was used as an outgroup. *Pseudomonas capsici* strains whose genome has been sequenced are labeled in red. Bootstrap values >70% are shown as percentages of 1,000 replicates. The scale bar represents nucleotide substitutions per site. Isolation host, location, year, *gryB* accession, and *rpoD* accession are shown. *Pseudomonas capsici* strains characterized in this study are labeled in red.

A phylogenetic analysis was conducted using the concatenated sequences of *gyrB* and *rpoD*, which had a total length of 961 nucleotides. Fifteen validly described *Pseudomonas* type strains were included in the analysis, with *P. graminis* DSM 11363^T^ serving as an outgroup. The resulting phylogenetic tree showed that the strains claimed to be a new phylogroup of *P. cichorii*, as well as 12 *P. capcisi* strains whose genomes were sequenced in this project including *P. capsici* type strain Pc19-1^T^, clustered together and formed a separate clade from the *P. cichorii* clade (Figure 2). This clade included *P. capsici* strains from diverse crops and diverse geographical locations. Additionally, the Georgia tomato strains Pc20-2, Pc20-3, and Pc20-4 were clustered together with the 17 Florida tomato strains isolated in 2012 (Figure 2). Pepper strains from Georgia were clustered together and close to tomato strains.

### 3.4. Host range

The host range of twelve *P. capsici* strains was evaluated on seven different plant species, including pepper cv. Aristotle, tomato cv. Glacier, Chinese cabbage cv. Rubicon, eggplant cv. Nadia, lettuce cv. Dragoon, broccoli cv. Arcadia F1, and endive cv. Curlesi. All twelve strains induced symptoms on the leaves of pepper, tomato, Chinese cabbage, eggplant, and lettuce, but did not cause any symptoms on broccoli and endive (Table 5). The symptoms observed on the tested hosts were more or less similar with initial water-soaked lesions that gradually turn necrotic. Control plants of each respective host did not exhibit any symptoms. The responses of seedlings were consistent across replicates and experiments. The isolates obtained from artificially inoculated plants were confirmed to be identical to the original inoculated strain through BOX-PCR analysis for DNA fingerprinting comparisons.

**Table 5:** Host range test results of *Pseudomonas capsici* strains.

Strain	Pepper (cv. Aristotle)	Tomato (cv. Glacier)	Chinese cabbage (cv. Rubicon)	Eggplant (cv. Nadia)	Lettuce (cv. Dragoon)	Broccoli (cv. Arcadia F1)	Endive (cv. Curlesi)
Pc19-1^T^	+^a^	+	+	+	+	–^b^	−
Pc19-2	+	+	+	+	+	−	−
Pc19-3	+	+	+	+	+	−	−
Pc20-2	+	+	+	+	+	−	−
Pc20-3	+	+	+	+	+	−	−
Pc20-4	+	+	+	+	+	−	−
Pc20-5	+	+	+	+	+	−	−
GEV417	+	+	+	+	+	−	−
GEV1127	+	+	+	+	+	−	−
NCPPB1511	+	+	+	+	+	−	−
NCPPB2479	+	+	+	+	+	−	−
NCPPB3928	+	+	+	+	+	−	−

a + indicates symptom development under greenhouse conditions.

b − plants did not show symptoms under greenhouse conditions.

### 3.5. Copper tolerance determination

**Figure 3:**
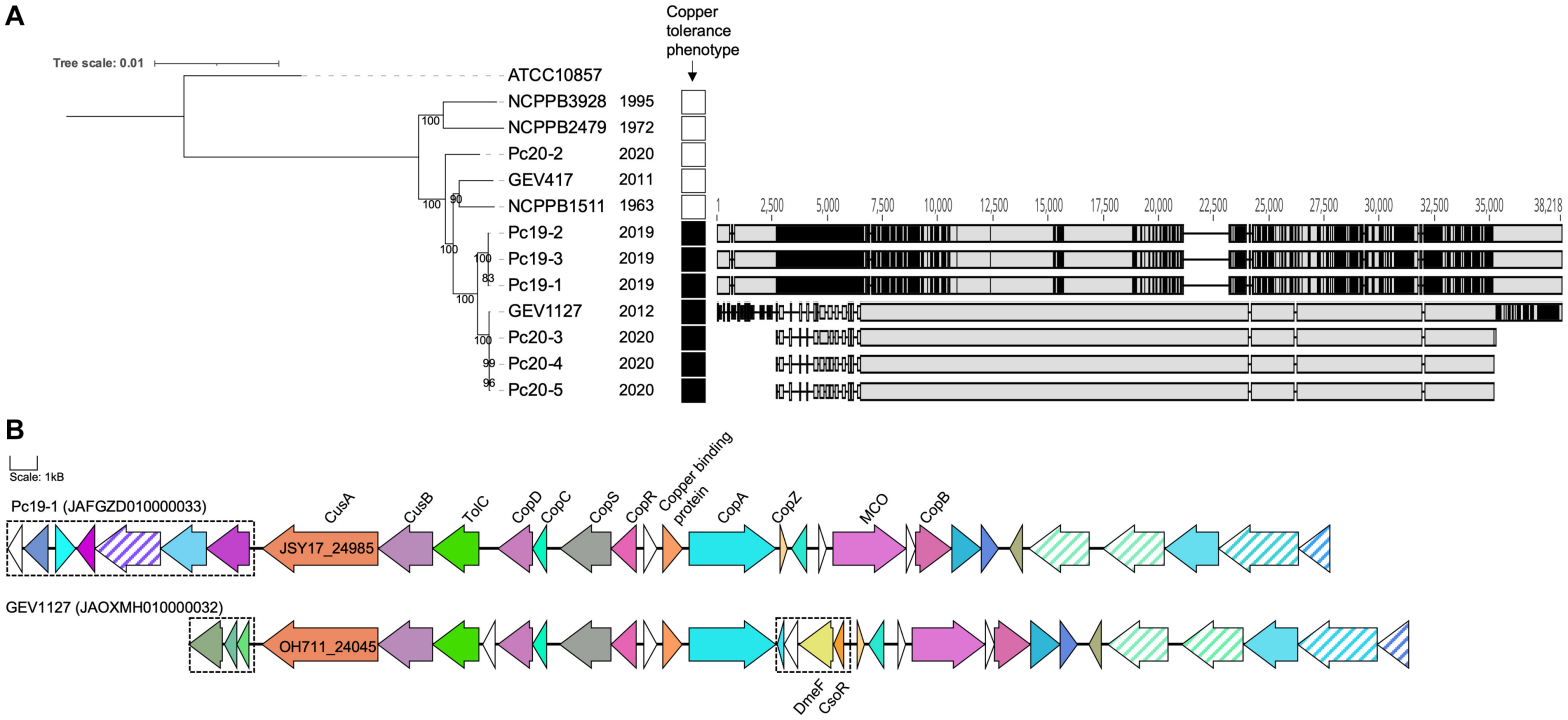
*Pseudomonas capsici* strains from 2012–2020 are copper tolerant. **(A)** A maximum likelihood phylogenetic tree based on whole-genome sequences of *P. capsici* strains and *P. cichorii* ATCC10857^T^ (as an outgroup). The bootstrap values are shown at the node. Isolation years are shown next to the strain name. Strains that showed growth on medium amended with 0.8 mM CuSO_4_ were annotated with black squares. The nucleotide alignment of copper-tolerant gene clusters unique in the copper tolerant strains was made using MAFFT in Geneious and shown on the right. Grey color indicates homology, and black color indicates single nucleotide polymorphism positions. **(B)** The operon maps of the copper tolerant gene clusters unique in *P. capsici* copper-tolerant strains. Copper tolerance-related gene annotations are labeled. The same colors represent homologous proteins. Transposase genes are in stripes. Differential genes between the clusters of Pc19-1 and GEV1127 are in dashed boxes. The scale represents 1 kB.

All 12 tested *P. capsici* strains were able to grow on NA medium that was not amended with CuSO_4_· 5H_2_O. Out of the 12 strains, seven strains were able to grow on NA medium amended with 0.8 mM CuSO_4_· 5H_2_O. However, the remaining five *P. capsici* strains were not able to grow on the same copper amended medium. Analysis of the copper-tolerance related subsystems in RAST revealed that copper tolerance gene clusters are present only in seven copper-tolerant strains (Pc19-1, Pc19-2, Pc19-3, GEV1127, Pc20-3, Pc20-4, and Pc20-5), but are absent in the remaining five strains (NCPPB3928, NCPPB2479, Pc20-2, GEV417, and NCPPB1511). Interestingly, two types of copper tolerance gene clusters were observed in the seven copper-tolerant strains listed above (Figure 3). Three strains (Pc19-1, Pc19-2, and Pc19-3) had identical sequences, while the other four strains (GEV1127, Pc20-3, Pc20-4, and Pc20-5) had identical sequences for their respective aligned region (Figure 3). The pairwise identity of the copper tolerance gene clusters between Pc19-1^T^ and GEV1127 was 77.3%. The copper tolerance gene cluster in strain GEV1127 included four genes that were absent in the Pc19-1^T^ cluster, including a DUF1289 domain-containing protein, a hypothetical protein, a CDF family Co (II) / Ni (II) efflux transporter DmeF, and a metal/formaldehyde-sensitive transcriptional repressor (Figure 3). These copper tolerance gene clusters were not found in other publicly available *P. capsici* genomes.

### 3.6. Genome-enabled identification of putative virulence factors and secondary metabolite gene clusters

**Figure 4:**
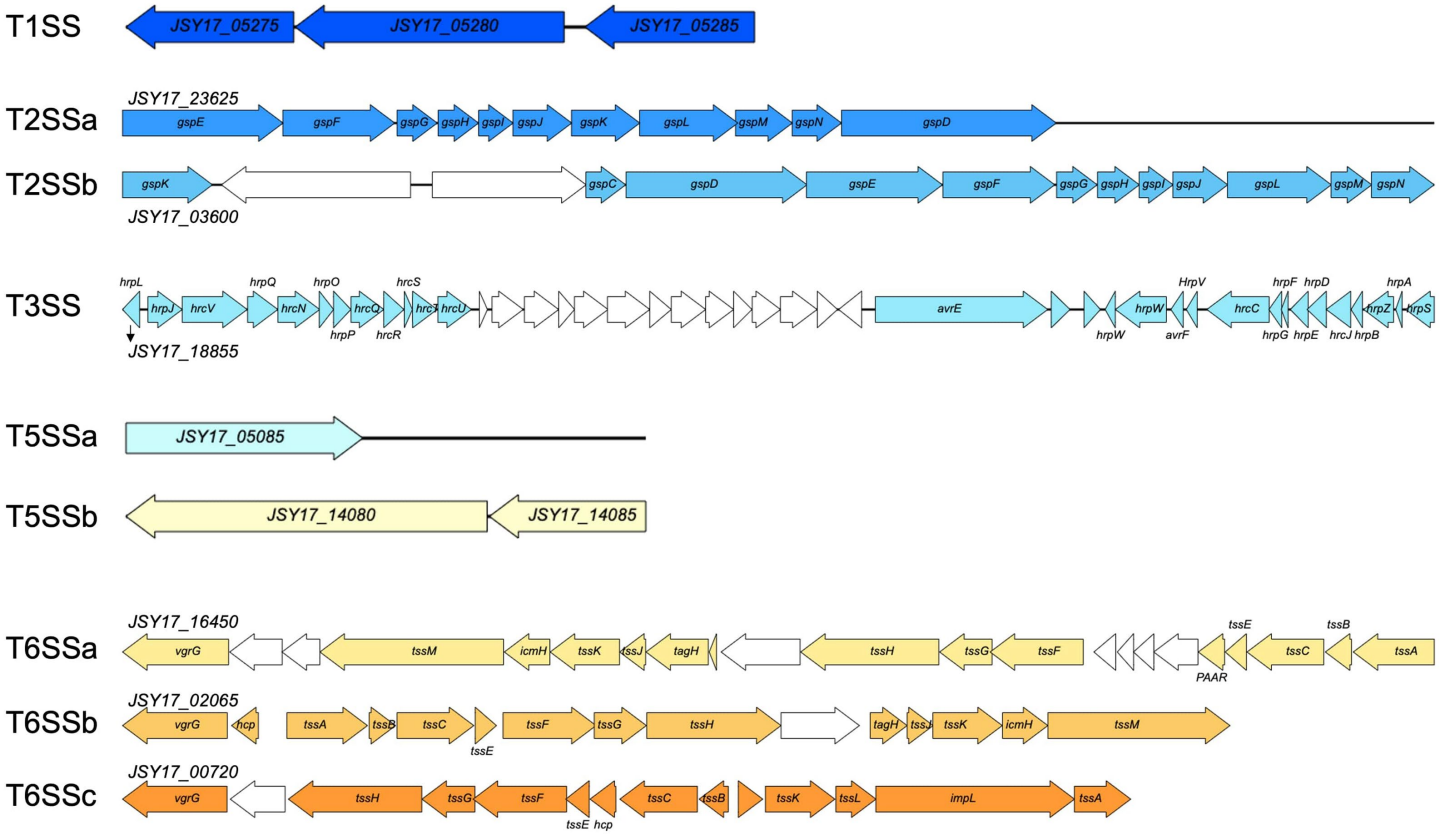
Gene synteny of secretion systems identified in *Pseudomonas capsici* strain Pc19-1^T^ genome. Colored arrows indicate secretion system related genes, and white arrows indicate genes annotated with hypothetical proteins or other functions.

The *P. capsici* strains possess an array of secretion systems, including one set of type I secretion system, two sets of type II secretion systems, one set of type III secretion system, two sets of type V secretion systems, three sets of type VI secretion systems (Figure 4). In addition, 10 gene clusters associated with secondary metabolites were identified using antiSMASH and are listed in Table 6. These gene clusters are linked to a variety of products, including a non-ribosomal peptide synthetase (NRPS) and type I polyketide synthase product (similar to yersiniabactin), an NRPS product (similar to cichopeptin), an NRPS product (similar to pyoverdine), an NRPS beta-lactam product (similar to thanamycin), an aryl polyene product, an N-acetylglutaminylglutamine amide product, an NRPS lipopeptide product (similar to cichofactin), a type III polyketide synthase product (similar to fisherindole), a redox-cofactor type product (similar to lankacidin C), and an NRPS product (similar to fragin).

**Table 6:** Secondary metabolite biosynthetic gene clusters in *Pseudomonas capsici* strains predicted by antiSMASH v6.

Type	Predicted product	Most similar known cluster
NRPS^a^, T1PKS^b^	NRP + Polyketide	Yersiniabactin
NRPS	NRP	Cichopeptin
NRPS	NRP	Pyoverdin
NRPS	NRP: Beta-lactam	Thanamycin
arylpolyene	other	APE Vf
NAGGN^c^	–^e^	–
NRPS	NRP: Lipopeptide	Cichofactin A/ Cichofactin B
T3PKS^d^, NRPS-like T1PKS	Alkaloid	Fisherindole
redox-cofactor	NRP + Polyketide	Lankacidin C
NRPS	NRP	Fragin

a NRPS: Non-ribosomal peptide synthetase cluster.

b T1PKS: Type I PKS (Polyketide synthase).

c N-acetylglutaminylglutamine amide.

d T3PKS: Type III PKS.

e −: no information available in the antiSMASH prediction output.

## 4. Discussion

The genus *Pseudomonas* is highly diverse, and its taxonomy has undergone many changes over the years. Within the *P. syringae* species complex, 13 distinct phylogroups have been identified based on the use of multilocus sequence analysis. *Pseudomonas cichorii* is a bacterial pathogen that can infect a wide range of crops, leading to significant yield losses in commercial agriculture. This pathogen is known to cause leaf spot, blight, and wilting in infected plants (Wilkie and Dye, 2012; Gappa-Adachi et al., 2014; Wang et al., 2022). Phylogenetic analysis based on *gyrB* and *rpoD* has placed *P. cichorii* within the *P. syringae* complex (Yamamoto et al., 2000) and was later grouped in phylogroup 11 based on multilocus sequence analysis of four housekeeping genes (Berge et al., 2014). In a study by Timilsina et al. (2017), tomato strains from Florida were identified as a novel phylogenetic group of *P. cichorii*. However, our analysis based on dDDH and ANIb values, as well as phylogenomic analysis, revealed that the previously reported Florida *P. cichorii* strains GEV417 and GEV1127 were actually *P. capsici*. Recent advances in high-throughput sequencing technologies have greatly facilitated the analysis of whole-genome sequences of bacterial strains, enabling researchers to infer evolutionary and taxonomic relationships (McAdam et al., 2014). Our analysis has also revealed inaccuracies in the classification of 13 strains in NCBI (473, MAFF 302698, Ku1409-10-1, NB15027, LCDW06, LCFQ22, 481, 482, 136, 474, WSXC14, s-2-2-1, and ICMP 1649), underscoring the importance of prudent species identification. Other examples of error in species designation of *Pseudomonas* strains are described here. The strain PPST 50936 from stevia (*Stevia rebaudiana*) in Florida was initially identified as *P. cichorii* based on the LOPAT scheme and sequence similarity (Strayer et al., 2012). Similarly, strains (14-WTREC and TN-E4) from pumpkin (*Cucurbita pepo* L. var. pepo) in Tennessee were identified as *P. cichorii* based on the LOPAT scheme (Newberry et al., 2016). However, our *gyrB-rpoD* tree analysis indicated that these strains were more closely related to *P. capsici* than *P. cichorii* (Figure 2). Therefore, it is likely that these strains may potentially be *P. capsici.* However, without the whole genome-based dDDH and ANIb analysis, it is difficult to deduce species designation.

Through phylogenomic and phylogenetic analyses (Figures 1, 2), it was discovered that *P. capsici* strains, despite being newly described, exhibited a broad host range. This was evidenced by their isolation from various sources such as lettuce, Chinese cabbage, coffee (*Coffea arabica*), tomato, soybean (*Glycine max*), wild soybean (*Glycine soja*), chrysanthemum (*Chrysanthemum* sp.), tobacco (*Nicotiana tabacum*), wild celery (*Apium graveolens*), pepper, cabbage (*Brassica oleracea*), stevia, hibiscus (*Hibiscus* sp.), basil (*Ocimum basilicum*), *Ficus pandurata*, pumpkin, *F. lyrata*, and crown of thorns (*Euphorbia milii*). Furthermore, the results of our artificial inoculation assays revealed that 12 *P. capsici* strains tested were pathogenic on pepper, tomato, Chinese cabbage, eggplant, and lettuce, but non-pathogenic on broccoli and endive. These observations indicate a diverse host range for *P. capsici* strains across different plant families, which may have practical significance in choosing crops for rotation or for companion cropping systems. However, it is interesting to note that although *P. capsici* is closely related to *P. cichorii* it is not pathogenic on endive, which is an original source of isolation for the *P. cichorii* type strain (ATCC 10857^T^).

Copper-based sprays have been extensively utilized in agriculture for many years for the control of bacterial and fungal diseases. As a result copper-tolerance have been reported in bacterial pathogens affecting multiple agricultural crops including pepper, tomato, and apple. The presence of these copper-tolerant bacterial strains reduces the efficacy of copper-based bactericides (Marco and Stall, 1983; Martin et al., 2004; Strayer-Scherer et al., 2018). Our research shows that all Georgia *P. capsici* strains from pepper and three out of four Georgia *P. capsici* strains from tomato exhibited copper tolerance *in-vitro*. The copper-tolerant strains possess at least two different types of copper-tolerant gene clusters, which were absent in the copper-sensitive *P. capsici* strains. The copper-tolerant strains were predominantly from the recent outbreaks of pepper and tomato in Georgia (2012, 2019, and 2020), but not from outbreaks in or before 2011. The acquisition of these clusters probably provided a selective advantage to these strains resulting in recent outbreaks that were difficult to manage with a standard copper-based spray program.

We also observed two distinct copper-tolerant gene clusters in seven copper-tolerant *P. capsici* strains. This may suggest that the sources of the copper tolerance in tomato and pepper strains are distinct. The genes in both clusters encode for efflux RND transporter permease subunit, efflux RND transporter periplasmic adaptor subunit, copper homeostasis membrane protein CopD, copper homeostasis periplasmic binding protein CopC, heavy metal sensor histidine kinase, heavy metal response regulator transcription factor, heavy metal translocating P-type ATPase, cation transporter, copper resistance system multicopper oxidase, and copper resistance protein B. The specific contributions of the individual genes within these clusters remain unclear. Further research is also necessary to understand the regulation of these genes and the distribution of these genes in general *P. capsici* populations.

The analysis of the whole genomes of *P. capsici* strains has revealed the presence of several virulence factors and secondary metabolites that may enable them to infect plant tissues and evade plant defense mechanisms. However, mere presence of these factors does not associate them with virulence unless they are validated using mutational and functional analysis. Future studies will focus on characterizing these potential virulence factors in pepper and tomato. The genus *Pseudomonas* is widely distributed and plays a crucial role in environmental processes (Cornelis, 2008). In addition to their ecological significance, *Pseudomonas* species are renowned for their ability to produce a vast array of biologically active secondary metabolites (Gross and Loper, 2009). The production of phytotoxins by members of the *P. syringae* species complex has been shown to contribute to virulence, including phytotoxins like syringomycin, syringopeptin, and cichofactin. We hypothesize that multiple virulence factors including the type III secretion system and cichofactin product may provide a competitive advantage for infecting diverse hosts. Future studies will focus on this aspect as well. Overall, this is the first study to characterize *P. capsici* strains genotypically and phenotypically from tomato and compared them with strains isolated from diverse hosts. Also, host-range, copper-tolerance and presence and absence of potential virulence factors in *P. capsici* were among some of the novel findings in this manuscript.

## 5. Conclusions

Our study experimentally demonstrated that *P. capsici* strains were pathogenic on pepper and tomato. The strains studied were pathogenic on multiple hosts under experimental conditions. Our findings indicate that this bacterium can potentially be a challenging plant pathogen to manage as it can infect a wide range of host plants and can potentially cause significant damage to agricultural crops. Although the potential mechanism of pathogen survival and dissemination are yet to be determined, it is imperative that future studies should focus on this aspect to assess risks of wide-spread outbreaks. To minimize the impact of *P. capsici*, it is essential that measures are taken to identify and exclude sources of inoculum, and monitor and control their reoccurrences. Furthermore, it is imperative that we continue to investigate the biology and disease cycle of this pathogen. This knowledge will help us to develop sustainable and effective management strategies that will aid in reducing losses due to this bacterial pathogen.

## Data availability statement

The datasets presented in this study can be found in online repositories. The names of the repository/repositories and accession number(s) can be found at: https://www.ncbi.nlm.nih.gov/genbank/, BioProject PRJNA890938.

## Author contributions

MZ: Conceptualization, Data curation, Formal analysis, Investigation, Methodology, Validation, Writing – original draft, Writing – review & editing. RG: Conceptualization, Writing – review & editing. BD: Conceptualization, Writing – review & editing, Data curation, Formal analysis, Funding acquisition, Investigation, Methodology, Project administration, Resources, Supervision, Validation, Visualization, Writing – original draft.

## Footnotes

^1^ https://www.bioinformatics.babraham.ac.uk/projects/fastqc/

^1^ https://microbializer.tau.ac.il/index.html
